# Microglia and astrocytes underlie neuroinflammation and synaptic susceptibility in autism spectrum disorder

**DOI:** 10.3389/fnins.2023.1125428

**Published:** 2023-03-20

**Authors:** Yue Xiong, Jianhui Chen, Yingbo Li

**Affiliations:** Cerebrovascular Diseases Laboratory, Institute of Neuroscience, Chongqing Medical University, Chongqing, China

**Keywords:** autism spectrum disorders, microglia, astrocytes, neuroinflammation, synaptic pruning, mitochondria, methylation

## Abstract

Autism spectrum disorder (ASD) is a common neurodevelopmental disorder with onset in childhood. The mechanisms underlying ASD are unclear. In recent years, the role of microglia and astrocytes in ASD has received increasing attention. Microglia prune the synapses or respond to injury by sequestrating the injury site and expressing inflammatory cytokines. Astrocytes maintain homeostasis in the brain microenvironment through the uptake of ions and neurotransmitters. However, the molecular link between ASD and microglia and, or astrocytes remains unknown. Previous research has shown the significant role of microglia and astrocytes in ASD, with reports of increased numbers of reactive microglia and astrocytes in postmortem tissues and animal models of ASD. Therefore, an enhanced understanding of the roles of microglia and astrocytes in ASD is essential for developing effective therapies. This review aimed to summarize the functions of microglia and astrocytes and their contributions to ASD.

## 1. Introduction

Autism spectrum disorder (ASD) is a common neurodevelopmental disorder, characterized by impaired social interaction, communication deficits, repetitive behavior, and narrow and intense interests (Kim et al., 2017). Autistic symptoms emerge in childhood and persist throughout life (Christensen et al., 2016). Although the precise etiologies of ASD are complex, recent evidence points to a contribution of glial cells in the pathophysiology of ASD. “Neuroglia” or “glia” include neuro-epithelial cells [oligodendrocytes (OLGs), astrocytes, oligodendrocyte progenitors, and ependymal cells], neural crest cells (peripheral glia), and myeloid cells (microglia) (Escartin et al., 2021). A recent study suggested that the activation of glial cells may contribute to the cognitive and behavioral impairments of ASD (Petrelli et al., 2016). Glial cells not only have neuronal “gluing” roles but are also involved in neurogenesis, synaptogenesis, inflammation, proper glutamate handling, and many other processes (Gzielo and Nikiforuk, 2021).

Microglia, as the resident macrophages in the central nervous system (CNS), act as the first main form of active immune defense in the brain and spinal cord (Kern et al., 2016; Umpierre and Wu, 2021). As the most abundant glial cells in the CNS, astrocytes play important brain functions in early development and adulthood, such as neurogenesis, synaptic development, synaptic transmission and plasticity, and regulate behavior under physiological and pathological conditions (Wang et al., 2021). Some transcriptions support an essential role in glial cell pathophysiology in the autistic brains. Gliosis and increased glial cell proliferation have been found in human postmortem brain samples (Petrelli et al., 2016). Moreover, glial abnormalities were found using animal models of ASD, such as Rett syndrome (RTT), Fragile X syndrome, and a mouse model of tuberous sclerosis.

This review discussed how microglia and astrocytes regulate the pathogenesis of ASD. In addition, the interaction between microglia and astrocytes in ASD was discussed. Finally, we describe the possible involvement of the mitochondria and methylation in regulating ASD by microglia.

## 2. Microglia contribute to neuroinflammation in ASD by releasing cytokines to prune synapses

As resident immune cells in the CNS, microglia, which express Iba1, Cx3cr1, CD11b, and F4/80, are the primary mediators of neuro-inflammation (Cowan and Petri, 2018; Hickman et al., 2018). They appear on embryonic day eight and mature 2–3 weeks after birth. Their morphology could change from immature amoebae to mature ramify, maintain tissue homeostasis, and exert innate immune functions through their multiple unique phenotypes and ability to transfer functions (Gzielo and Nikiforuk, 2021). Reactive microglia have neuroinflammatory and neuroprotective properties (Voet et al., 2019). Mediators derived from mast cells could activate microglia, causing localized inflammation and leading to symptoms of ASD (Zhang et al., 2012; Skaper et al., 2017; Kempuraj et al., 2019). Microglia could be divided into two activation states: M1 type (classical activation) and M2 type (alternative activation) (Orihuela et al., 2016; Liao et al., 2020). The M1 phenotype microglia produce inflammatory cytokines and reactive oxygen species (ROS), and the M2 phenotype microglia produce anti-inflammatory cytokines and neurotrophins. Microglia rely on CSF1 and transcription factors, such as interleukin (IL)-34 and IRF8 for survival and maintenance (Hickman et al., 2018). They also induce the expression of target inflammatory genes through different signaling pathways, such as JNK, JAK/STAT, ERK1/2, NF-kB, and p38 (Zhang et al., 2012; Skaper et al., 2017; Kempuraj et al., 2019; Voet et al., 2019).

Microglia are indispensable regulators of inflammatory responses in the CNS (Kwon and Koh, 2020). Under physiological circumstances, microglia exert highly efficient surveillance mechanisms to clear invading pathogens and promote tissue repair. Under pathological conditions, the developing brain is very sensitive to environmental stimuli, and it produces a robust inflammatory response that leads to neuroinflammation, in which microglia react (gliosis), proliferates, and recruits peripheral blood white blood cells, thereby amplifying the initial tissue damage, meanwhile, reactive gliosis may exacerbate the inflammatory state caused by immune activation involved in the pathogenesis of ASD (Petrelli et al., 2016). In the present study, the association between reactive microglia and neuroinflammatory responses in ASD was discussed.

### 2.1. Cytokines and chemokines released by microglia regulate neuroinflammation

Microglia are activated in multiple brain regions of young adults with ASD by functional positron emission tomography (PET) imaging (Petrelli et al., 2016). Increased pro-inflammatory cytokines in blood and cerebrospinal fluid (CSF) and increased microglia number and activation in the postmortem dorsolateral prefrontal cortex (DLPFC) provide strong evidence of neuroinflammation in ASD (Zantomio et al., 2015). In addition, changes were observed in the expression levels of pro-inflammatory (CD68 and IL-1β) and anti-inflammatory genes (IGF1 and IGF1R) in gray- and white-matter tissues of ACC in males with ASD (Sciara et al., 2020). Current studies have shown that the gene expression of anti-inflammatory cytokine IL-37 and pro-inflammatory cytokines IL-18 and TNF increases in the amygdala and dorsolateral prefrontal cortex of children with ASD (Tsilioni et al., 2019). In addition, IL-38 is decreased in the amygdala of children with ASD (Tsilioni et al., 2020).

Chemokines, as a subset of cytokines that guide cell migration, are mainly divided into two categories: CXC chemokines and CC chemokines (including CCL2 (MCP-1), CCL3 (MIP-1α), CCL4 (MIP-1β), CCL5 (RANTES), CCL11 (Eotaxin)). CCL2 is conformably elevated in the brain and blood of individuals with autism and has been extensively studied. CCL2 is produced by microglia and astrocytes in the CNS, and in turn, CCL2 regulates the reactivity, migration, and proliferation of microglia (Matta et al., 2019; Ye et al., 2021). In the offspring of maternal exposure to CAF (cafeteria diet) diet or Poly (I: C) inoculation, CCL2 signaling disrupts social behavior by microglia morphology (Maldonado-Ruiz et al., 2022). Flavonoid methoxy luteolin, a peptide neurotensin (NT) inhibitor, reduced the gene expression and release of proinflammatory cytokines IL-1β, CCL2, and CCL5 in human microglia (Patel et al., 2016). All data support cytokines and chemokines as essential mediators in neuroinflammation and autism-like behaviors (Table 1).

**Table 1:** Variations of cytokine and chemokines in ASD.

	Factor	CSF	Whole blood serum
**Cytokine**			
	TNF-α	Increased	Increased
	IL-6	Increased	Increased
	IL-17	Increased	Increased
	IL-1β	Increased	Increased
**Chemokines**			
	CCL2	Increased	Not reported
	CCL3	Not reported	Not reported
	CCL4	Not reported	Not reported
	CCL5	Not reported	Not reported
	CCL11	Not reported	Not reported

CSF, cerebrospinal fluid.

### 2.2. Microglia prune synapses by phagocytosis and elimination

Microglia are involved in the development of excitatory circuits through engulfing and eliminating of synapses, called “pruning” (Lewis, 2021). ASD is often accompanied by abnormalities in synapses. Evidence showed increased density of dendritic spines and abnormal synaptic structure in the brains of ASD model mice (Kim et al., 2017). Microglia shape synaptic function and plasticity through dynamic morphological and functional properties (Ben Achour and Pascual, 2010). Synaptic phagocytosis by microglia is one of the most intensively studied methods to regulate synaptic plasticity. Presynaptic and postsynaptic components within microglial lysosomes have been identified by electron microscopy and high-resolution *in vivo* imaging (Paolicelli et al., 2011; Sancho et al., 2021). The complement cascade is one of the classical phagocytosis pathways mediated by microglia, in which complement component 1q (C1q) initiates C3 on neurons to bind complement receptor 3 (CR3) on microglia to target phagocytic synapses (Sancho et al., 2021). In addition, microglia could remove synapses by phagocytosis *via* the CX3C chemokine receptor 1 (CX3CR1) and CR3 pathways (Li et al., 2020). However, microglia could shape neuronal connectivity though non-phagocytic mechanisms. Postsynaptic calcium elevation increases the likelihood of dendritic spine formation due to microglial contact with dendrites. Conversely, microglial contact with dendritic spines could also increase the possibility of spine retraction by modifying the local extracellular matrix and reducing synaptic stability. Dendritic spine dynamics and synaptic AMPAR transport could be influenced by BDNF and TNFα, respectively, secreted by microglia, which perform their function of partially encapsulating synapses rather than engulfing them (Blagburn-Blanco et al., 2022).

In particular, microglia clustered around neurons in the dorsolateral PFC of patients with ASD due to alterations in the spatial structure of microglia (Varghese et al., 2017). On the one hand, microglia constantly palpate the neuronal surface (Bal-Price and Brown, 2001). In a mouse model of PTEN localization in the cytoplasm (Ptenm3m4/m3m4), evidence of cross-communication between neurons and microglia was found, with Ptenm3m4/m3m4 neurons inducing enhanced pruning from naturally activated microglia (Sarn et al., 2021). In primary cultures of rat microglia and neurons, carbon monoxide exerts antineuroinflammatory and neurotrophic effects by regulating microglia–neuron communication (Soares et al., 2022). On the other hand, microglia could cause the death of phagocytosed cells by engulfing live neurons and neuronal progenitors. Changes in the activation state of microglia affect brain development, possibly through the uptake of neural precursor cells by phagocytosis (Brown and Neher, 2014). The phenomenon is mainly divided into “eat-me” and “do not-eat-me.” When microglia detect exposed “eat-me” signals, they rapidly recognize and phagocytose neurons or parts of neurons exposed to the signal. In performing phagocytosis, the “do not-eat-me” signal occurs when inhibitory neuron cell surface signals are absent or removed. Phospholipid phosphatic glycerine is a key “eat-me” signal for microglia to phagocytize dead and surviving neurons. Plasminogen activator inhibitor type 1 (PAI1) acts as a “do not-eat-me” signal on neutrophils, inducing microglial migration but also inhibiting VNR-mediated microglial phagocytosis (Brown and Neher, 2014). *In vitro*, microglial inflammation is activated by TNF-α, Toll-like receptor ligand (TLR), or amyloid-β. Upon activation, microglia release sublethal amounts of reactive nitrogen (RNS) and ROS, leading to reversible phosphatidylserine orientation on neurons and thus triggering microglial phagocytosis of them. When agents are not enough to kill neurons directly, they may induce exposure and, or release molecules (UDP, phosphatidylserine, and calreticulin) by exerting sufficient stress on neurons, triggering microglia phagocytosis in stressed but surviving neurons and eventually leading to cell death by phagocytosis (Fricker et al., 2018).

Microglia play a role in ASD by participating in synaptic pruning. Some animal studies provide strong proof. For example, germline mutations in the tumor suppressor gene *PTEN* are one of the monogenic risk cases for ASD. By generating a nuclear-predominant PtenY68H/+ mouse model, prominent reactive microglia were found, accompanied by enhanced phagocytosis (Sarn et al., 2021). Furthermore, deletion of atg7 was shown to cause autism-like behavior in a myeloid cell-specific lysozyme M-Cre mouse model. Then, co-culture with AtG7-deficient microglia impaired synaptosome degradation and increased immature dendritic filopodia (Kim et al., 2017).

TREM2 is involved in the phagocytosis of excess synapses in the CA1 region of the mouse hippocampus during development (Sancho et al., 2021). TREM2^−/−^ mouse models typically displayed altered sociability and repetitive behavior. TREM2 protein levels were often negatively correlated with the severity of symptoms in patients with ASD (Filipello et al., 2018). Neuronal defects caused by Hoxb8-microglial defects and mutations in synaptic components could cause mice to exhibit autism-like behavior (Nagarajan et al., 2018). A mouse model lacking CX3CR1 showed a transient decrease in microglia and a consequent defect in synaptic pruning during the early postnatal period. In a mouse model of microglial Tmem59 deletion, deletion of microglial Tmem59 impaired synaptic phagocytosis, leading to autism-like behavior (Meng et al., 2022). In autism models, a transient decrease in microglia is followed by a synaptic pruning defect, strongly associated with autistic behaviors such as social deficits. These findings further confirmed that disrupted synaptic pruning mediated by microglia might contribute to ASD (Zhan et al., 2014). Microglia play a unique role in establishing and maintaining the delicate balance of excitatory and inhibitory synapses. Dysfunctional social and cognitive behavior was demonstrated to be associated with alterations in excitatory and inhibitory synaptic connections in the mPFC in ASD. In addition, reductions in mPFC spine density have been described in mouse models of ASD. More importantly, inhibitory neuronal function and synapses are modulated by specific ASD risk genes. For example, most of the behavioral features of RTT were reproducible when the risk MECP2 gene was deleted from all GABAergic interneurons (Blagburn-Blanco et al., 2022).

### 2.3. Microglia modulate the excitatory/inhibition balance in ASD by pruning synapses

Evidence showed that glial cell function is related to an imbalance between excitatory and inhibitory synaptic function (Andoh et al., 2019a,b). The structural and functional breakdown of the balance between E/I synapses is the pathogenesis of CNS diseases. After aberrant synaptic pruning in microglia was discussed, microglial synaptic pruning resulting in synaptic excitatory to inhibitory (E/I) imbalance was explored (Andoh et al., 2019a,b). Neurons receive excitatory and inhibitory inputs and maintain a balance between the two, known as the E/I balance. If the E/I balance is disrupted, such as increased levels of excitatory input, associated with autism, it could affect brain function and social behavior (Pretzsch and Floris, 2020). To date, studies on microglia-mediated synaptic pruning have focused on excitatory synapses (Favuzzi et al., 2021). Microglia participate in glutamate signaling through the Xc system, and the Xc transporter in the Xc system is a chloride-dependent antiporter that could carry glutamate out of the cell. Microglia produce ROS, which induces glutathione (GSH) deficiency and initiates the TLR4 signaling pathway, causing an increase in Xc expression and resulting in glutamate efflux. The ROS, IL-1β, and TNF-α secreted by microglia impair EAAT function and increase extracellular glutamate levels. In summary, reactive microglia actively interfere with neurotransmission through the impaired glutamate uptake; release of excitotoxins, such as glutamate, D-serine, and ATP; and alteration of glial transmitter release from astrocytes (Kim et al., 2020). Whether microglia also actively shape the developing inhibitory circuit is not known. However, during development, GABA-receptive microglia selectively prune inhibitory synapses, presenting behavioral abnormalities due to disruption of microglial responses, thus highlighting a critical function of microglia-mediated inhibitory synaptic pruning (Favuzzi et al., 2021).

The autism-like phenotype could be altered by altering the excitation–inhibition balance between microglia and astrocytes. Collectively, the present study demonstrated associations between changes in microglia and E/I balance in ASD.

### 2.4. Sex differences in microglia may underlie ASD susceptibility

The salience network (SN), central executive network (CEN), and default mode network (DMN) are central to ASD symptomatology. Gender differences exist in the functional connectivity of SN, CEN, and DMN in adolescents with ASD. Therefore, sex-specific biological factors should be considered when investigating the neural mechanisms of ASD (Lawrence et al., 2020). ASD is well known to be approximately four times more common in males than in females. The mechanisms underlying this sex-differential risk are not fully understood, making it more difficult to study the mechanisms behind the risk of gender differences in ASD.

Microglia play an important role in the sex difference in ASDs (Schwarz and Bilbo, 2012; Andoh et al., 2019a,b). For example, high expression levels of microglia markers were observed in males (Werling et al., 2016). In a model of exposure to low lead concentrations during pregnancy, increased glial cells proliferation in the cerebellum of lead-exposed male pups led to an increased incidence of autism-like behavior, suggesting that sex-dependent glial cells influence the incidence of autism-like behavior (Choi et al., 2022). Further evidence regarding sex-specific differences in microglia could be found. For example, a genome-wide association study (GWAS) provided evidence of the upregulation of genes, including microglia markers found in the postmortem brains of male patients with ASD (Werling et al., 2016). In addition, one study showed that exaggerated translation of only microglia caused autism-like behavior in male mice (Xu et al., 2020). In conclusion, sex differences in microglia may underlie vulnerabilities to ASD.

## 3. Neurotransmitter and ion channels expressed by astrocytes facilitate communication between astrocytes and synapses

The immune function of astrocytes is similar to that of microglia (Sofroniew, 2015). From postnatal day 14 to postnatal day 30, astrocytes develop from initial maturation to full maturation (Gzielo and Nikiforuk, 2021). Astrocytes become reactive astrocytes after injury (Kim and Son, 2021). Reactive astrocytes establish immune responses through morphological changes and proliferation. The process achieved is through extension and hypertrophy, distinct from the microglial contraction process. Astrocytes are divided into A1 neurotoxic phenotype and A2 neuroprotective phenotype (Escartin et al., 2021). The A1 astrocyte phenotype is generated by microglia stimulated by lipopolysaccharide (LPS) *via* TNF, IL-1α, and C1q (Giovannoni and Quintana, 2020). In addition, a soluble neurotoxin secreted by A1 astrocytes could quickly kill neurons and mature OLGs. By contrast, A2 astrocytes have repair functions and could upregulate neurotrophic or anti-inflammatory genes to promote neuronal survival and growth. The most generally commonly used specific markers for A1 and A2 astrocytes were C3, S100a10, and PTX3 (Li et al., 2020; Fan and Huo, 2021). Reactive astrocytes have not only harmful effects of aggravating neuro-inflammation and hindering synaptic sprouting or axon growth but also beneficial effects of anti-inflammation, neuroprotection, and blood–brain barrier repair (Hickman et al., 2018; Fan and Huo, 2021).

Sixty-five percent of the 46 most significant autism-associated genes are expressed in astrocytes, according to a recent GWAS analysis (Yu et al., 2013). Astrocytes are found to be activated in those with ASD diagnosis. The expression of a glial fibrillary acidic protein (GFAP) is upregulated when astrocytes are hypertrophic and proliferate, and in children diagnosed with ASD, GFAP levels were found to be three times higher than controls in the brain and CSF (Kern et al., 2016). In addition, the number of GFAP-positive cells changed in a VPA and poly (I: C) model (Zhao et al., 2019; Gzielo and Nikiforuk, 2021). In a 35-day-old VPA rat model, studies have shown that GFAP immunostaining levels were increased in the medial prefrontal cortex and hippocampus (Mony et al., 2018). In addition to GFAP, samples of patients with ASD showed abnormal expression of astrocyte markers AQP4 and CX43 (Sloan and Barres, 2014).

Astrocytes cannot only regulate inflammation but also maintain homeostasis within the brain by modulating synaptic function and plasticity (Matta et al., 2019). In addition, A1 reactive astrocytes induced the formation of fewer synapses than synapses generated by healthy quiescent astrocytes (Liddelow et al., 2017). Microglia have brief periodic contact with synapses, and astrocytes are conversely warped around pre- and post-synapses as part of the tripartite synapse (Matta et al., 2019; Gzielo and Nikiforuk, 2021). A large number of receptors, adhesion molecules, and ion channels are distributed around astrocyte synapses, and they are essential for maintaining synaptic function (Gzielo and Nikiforuk, 2021).

### 3.1. Astrocytes regulate neurotransmitter homeostasis in ASD

E/I neurotransmission imbalance is involved in the pathogenesis of ASD, mainly by altering glutamatergic and GABAergic neurotransmission (Canitano and Palumbi, 2021). Astrocytes regulate neurotransmitter homeostasis in the CNS by uptaking synaptically released neurotransmitters, such as glutamate, glycine, and γ-aminobutyric acid (GABA), and releasing their precursors back to neurons after metabolism (Sofroniew and Vinters, 2010). Glutamate is one of the most prevalent universal neurotransmitters released by excitatory neurons in the CNS (Doughty et al., 2020). Astrocytes maintain glutamate homeostasis and prevent glutamate excitotoxicity by controlling the balance of glutamate release and uptake (Mahmoud et al., 2019). The main pathway of glutamate uptake is achieved by two glutamate transporters: Na^+^-dependent and -independent transporters (Anderson and Swanson, 2000; Mahmoud et al., 2019). Several studies have provided evidence of changes in astrocyte glutamate in ASD. In a VPA-induced ASD rat model, an increase in glutamate uptake was found at postnatal day 120 (Bristot Silvestrin et al., 2013). In a ^1^H-MRS model of children with ASD, abnormalities of glutamate metabolites in the anterior cingulate cortex (ACC) were observed through brain functional magnetic resonance imaging (Jiménez-Espinoza et al., 2021). Selective loss of astrocyte-specific Fmr1 knockout mice (i-Astro-Fmr1-cKO) and repair mice (i-Astro-Fmr1-cON) resulted in dysregulation of the glutamate transporter GLT1 and impaired extracellular glutamate uptake. Enhanced cortical neuronal excitability was also found in astrocyte-specific cKO mice (Higashimori et al., 2016). The glutamate transporter GLT1 is vital for regulating the E/I ratio in astrocytes. In an astrocyte-specific GLT1 knockout mouse model, the mice exhibited excessive repetitive behavior (Aida et al., 2015). However, in addition to the glutamate transport described above, glutamine synthetase (GS) also supports the amino acid neurotransmitter cycle. The glutamine used by neurons is dependent on the GS conversion of glutamate. Studies have shown that GABAergic neurons are more dependent on astrocyte glutamine than excitatory neurons, so the lack of astrocyte GS may lead to altered inhibitory neuronal function (Gzielo and Nikiforuk, 2021).

GABA, as a highly representative inhibitory neurotransmitter, regulates the overall functions controlled by the brain, such as the regulation of learning and memory functions. Impaired GABA transmission may be one of the pathological evidence of E/I imbalance. Astrocytes express GABA receptors (GABAR), mainly ionic GABAA and metabolic GABAB receptors, and GABA transporters (GATs), including GAT-1 and GAT-3. Previous studies have reported reductions in GABAergic interneurons and transmission in mouse models of ASD (Kim et al., 2020). Furthermore, in a model of maternal lead exposure, astrogliosis was able to prevent behavioral changes by ensuring high GABA levels (Choi et al., 2022). Meanwhile, inhibition of abnormally elevated GABAergic synaptic transmission in the hippocampal CA1 region has been shown to restore E/I balance and rescue autism-like behavior (Chen et al., 2022). Furthermore, attention was improved, and behavioral hyperactivity was alleviated in mice due to the inhibition of the astrocyte GABAB-Gi pathway in the striatum (Nagai et al., 2021).

### 3.2. Astrocytes regulate ion channels in ASD

Astrocytes are activated by ion (calcium, sodium, and potassium) transport and are not electrically excited (Gzielo and Nikiforuk, 2021). One of the critical functions of astrocytes is ion homeostasis. Fluctuations in intracellular ion concentration could mediate astrocyte excitability (Kirischuk et al., 2012). Glutamate release from astrocytes is achieved by the elevation of [Ca^2+^]i in astrocytes (Parpura et al., 1994; Bezzi et al., 1998). Ca^2+^ signaling is thought to underlie essential physiological functions of astrocytes in various species, such as worms, flies, zebrafish, mice, and possibly humans (Yu et al., 2020). Elevations in astrocytes’ Ca^2+^ could cause the release of gliotransmitters, glutamate, GABA, adenosine triphosphate (ATP), and D-serine, which could all modulate postsynaptic neuronal activity and act on presynaptic receptors. Ca^2+^ waves could propagate vasoactive messengers to the soma and its vascular endfeet through astrocytes (Bazargani and Attwell, 2016). In inflammation, the disruption of astrocyte calcium signaling is important (Allen et al., 2022). However, whether astrocytes play a mechanistic role in ASD through Ca^2+^ signaling remains unclear. In astrocyte-specific inositol 1,4,5-triphosphate six receptor type 2 (IP3R2) knockout mice and IP3R2-null mutant mice, IP3R2 led to astrocyte activation through the release of intracellular Ca^2+^ stores. The results suggested that astrocyte dysfunction by Ca^2+^ ions is associated with ASD-like phenotypes (Wang et al., 2021). In addition, astrocytes from individuals with ASD alter behavior and disrupt neuronal activity through abnormal Ca^2+^ signaling (Allen et al., 2022). All the evidence provides that Ca^2+^ signaling has critical physiological functions in ASD. Therefore, calcium signaling-induced changes in astrocytes could be an essential target for intervention in ASD.

Recent studies have shown that perisynaptic astrocyte cytosolic Na^+^ concentration ([Na^+^]i) could be triggered by neuronal activity, resulting in a transient increase. Na^+^-permeable channels and Na^+^-dependent transporters control [Na^+^]i transients, and astrocyte homeostasis responses are dynamically counter-regulated by [Na^+^]i. For example, [Na^+^]i transients dynamically regulate the transmembrane transport of neurotransmitters, the metabolism/signal utilization of lactate and glutamate, and K^+^ buffering (Kirischuk et al., 2012). Neurotransmitters, ion transport, amino acids, and many other molecules across the plasma membrane and inner membrane provide energy through an inwardly directed large sodium (+) gradient that puts sodium homeostasis at a central stage in astrocyte physiology. Na(+)/K(+)-ATPase (NKA), as the primary energy consumer of the brain, mediates Na(+) efflux from astrocytes, thereby maintaining Na(+) homeostasis (Rose and Verkhratsky, 2016). Available sodium channels and astrocyte expression have been confirmed by patch-clamp recordings. Importantly, Voltage-dependent sodium currents have been detected in astrocytes within the spinal cord and hippocampal slices (Pappalardo et al., 2016). Astrocytes buffer K^+^ by inward rectifying potassium channels (Kir) and aquaporin 4 (Aqp4) and regulating the flow of water and K^+^ between the extracellular space and neuronal cells, resulting in an imbalance between neuronal excitation and inhibition (Iliff et al., 2012; Gzielo and Nikiforuk, 2021). For example, impaired astrocyte K^+^ buffering, which results in increased neuronal excitation, is due to a loss of water channels, such as Aqp4, which underlies much of autism (Gzielo and Nikiforuk, 2021). In addition, riluzole, a sodium channel blocker, could effectively increase the inhibition index and normalize PFC functional connectivity in ASD (Sohal and Rubenstein, 2019). All the evidence shows the essential physiological functions of Na(+)/K(+) in ASD. Therefore, Na(+)/K(+) signaling-induced changes in astrocytes could be an essential target for intervention in ASD. In conclusion, astrocytes maintain the balance of cellular E/I ratio, thus promoting homeostasis in the CNS in ASD.

### 3.3. Astrocytes pruning synapses by expressing neurotransmitter receptors and transporters

Many neurotransmitter receptors and transporters expressed by astrocytes facilitate communication between astrocytes and synapses. For example, astrocytes could modulate synaptic transmission by inhibiting glutamate release from presynaptic neurons and altering receptor expression on postsynaptic neurons. They also trigger the phagocytic pathway through the expressing multiple epidermal growth factor-like domain protein 10 (MEGF10) and MER tyrosine-protein kinase (MERTK), thereby promoting synapse elimination. In addition, astrocytes indirectly trigger synapse elimination by secreting TGF-β, which induces C1q expression in retinal neurons to initiate microglia-mediated phagocytosis.

These findings suggested that astrocyte function may be relevant to the pathophysiology of ASD, such as its ability to influence neuronal circuits that are highly dynamic and plastic in the adult brain (Matta et al., 2019). Recent studies have found that astrocyte complement component 4 (C4) was significantly expressed in the anterior part of the human brain, the sub-ependymal zone (SVZ), and the surrounding area. Alternatively, the C4 protein was localized to neuronal cell bodies and synapses, suggesting that astrocytes may exert synaptic elimination effects through the C4 pathway (Mou et al., 2022).

Some evidence indicated reciprocal communication between astrocytes and neurons in -vitro and -vivo experiments. In a mutant RTT mouse model, the typical morphology of wild-type or mutant hippocampal neurons was disrupted by a vitro co-culture system of astrocytes (Ballas et al., 2009). Using pluripotent stem cells derived from non-syndromic ASD individuals, ASD-derived astrocytes were found to interfere with normal neuronal development through co-culture experiments (Russo et al., 2018). These findings further suggested that neuronal function may be affected by the inflammation of astrocytes (Lee et al., 2020).

## 4. The co-ordination of microglia and astrocyte modulates inflammation by the inflammatory mediator and secretion of multiple cytokines

Bidirectional communication exists between microglia and astrocytes, and it modulates CNS inflammation through the inflammatory mediator and secretion of multiple cytokines. In conclusion, the basis of neuronal function and dysfunction is microglia–astrocyte crosstalk (Jha et al., 2019). LPS-activated microglia induce reactive astrocytes (Liu et al., 2020), and, in turn, microglia are further activated by ATP released from reactive astrocytes (Traetta et al., 2021). LPS-activated microglia also induce a neurotoxic phenotype in reactive astrocytes. For example, recent studies have found that micro1glial cells secreting interleukins and chemokines, macrophage colony-stimulating factor (M-CSF), monocyte chemoattractant protein-1 (MCP-1), macrophage inflammatory protein-1α/β (MIP-α/β), TNF-α, and C1q could induce a transcriptional response in astrocytes, activating a neurotoxic factor that reduces the expression of neurotrophic factors (Zhang et al., 2010; Linnerbauer et al., 2020). In addition, microglia and astrocytes could be polarized into M2-type microglia and A2-type astrocytes, respectively, by *in-vitro* crosstalk (Kim and Son, 2021).

ORM2, a member of the lipocalin family expressed by astrocytes, regulates microglial activation in response to inflammatory stimuli. Astrocytic ORM2 could bind to the microglial C-C chemokine receptor type 5 (CCR5) and affect microglial activation by blocking the chemokine C-X-C motif ligand (CXCL)-4-CCR5 interaction, indicating the role of ORM2 in astrocyte–microglia interaction (Jo et al., 2017). In microglia–astrocyte co-cultures from VPA animals, microglia exhibited reactivity and exacerbated astrocyte reactivity (Traetta et al., 2021). Thus, the present study highlighted microglia–astrocyte communication as a novel mechanism of neuro-inflammation in ASD. Therefore, this crosstalk could be considered a potential target for intervention in ASD.

## 5. Mitochondria and methylation may be involved in the regulation of ASD by microglia

Mitochondria are dynamic organelles that undergo rapid changes in their structure and intracellular localization in the face of the needs of different cells (Ho and Theiss, 2022). One of the most common metabolic disorders in patients with ASD is abnormal mitochondrial function. In the latest study, PM2.5 exposure mediated through the mitochondria during gestation and early life could increase the risk of developing ASD (Frye et al., 2021a,b). Clinical epidemiological studies have demonstrated mitochondrial dysfunction in neurodevelopmental disorders (Thangaraj et al., 2018). Evidence suggests that mitochondrial DNA (mtDNA) is a major activator of inflammation when it leaks from stressed mitochondria (Zhong et al., 2019). Moreover, mtDNA escaping stressed mitochondria provokes inflammation *via* cGAS-STING pathway activation, and when oxidized (Ox-mtDNA), it binds to cytosolic NLRP3, thereby triggering inflammasome activation (Xian et al., 2022). In patients with myalgic encephalomyelitis/chronic fatigue syndrome (ME/CFS), who manifests with fatigue, malaise, sleep disorders, and cognitive problems, the exosome-associated mtDNA could stimulate human microglia to release IL-1β (Tsilioni et al., 2022). Moreover, mtDNA is significantly increased in the serum of children with ASD (Theoharides et al., 2013). In addition, FOXP1 syndrome, caused by haploinsufficiency of the forkhead box P1 (FOXP1) gene, is a neurodevelopmental disorder that manifests as motor dysfunction, intellectual disability, language impairment, and autism. Emerging evidence of mitochondrial dysfunction in FOXP1^+/−^ mice suggested that inadequate energy supply and excessive oxidative stress underlie cognitive and motor impairments caused by FOXP1 deficiency (Wang et al., 2022). In addition, odor identification impairment in ASD may be associated with mitochondrial dysfunction (Yang et al., 2022). The mitochondria are involved in astrocyte maturation and synapse formation. The microglia from embryonic ischemic cortical rats could proliferate by transplanting hamster mitochondria (Gyllenhammer et al., 2022). Therefore, mitochondrial dysfunction may play an important role in inducing glial abnormalities in autism.

DNA methylation has become an area of particular interest in ASD (Williams and LaSalle, 2022). Children with autism exhibit impaired methylation (Deth et al., 2008). Impaired methylation and epigenetic disruption contribute to the immune dysfunction commonly seen in autism (Deth et al., 2008). A study found that differentially methylated regions were enriched for transcription factor binding sites related to regulating microglial inflammation and microglial development (Vogel Ciernia et al., 2018). In brain cells, Methyl CpG binding protein-2 (MeCP2) isoforms (E1 and E2) are an important epigenetic regulator. MeCP2 loss- or gain-of-function mutation causes neurodevelopmental disorders, including ASD, MECP2 duplication syndrome, and RTT (Lioy et al., 2011; Liyanage et al., 2019). Studies from animal models of RTT and *MECP2* could explain the malfunction of epigenetic mechanisms in microglia. MeCP2 participates in the regulation of gene transcription by binding to methylated CG sites. A major study showed an RTT-like phenotype in microglia-specific MECP2 knockout mice that could be reversed by supplementation with wild-type microglia. In addition, the deletion of MECP2 in microglia was demonstrated to cause abnormalities in extracellular glutamate levels and neuronal dendrites. These studies suggested that MeCP2 influences mouse behavior by regulating the epigenetic machinery in microglia. Subsequently, other genes associated with ASD, OXTR, MAGEL2, SNRPN, RELN, and GAD1, were found to have hypermethylated transcription start sites in ASD brains, resulting in reduced expression of gene products (Tremblay and Jiang, 2019). While the exact role of microglia is not completely defined, much evidence could suggest that the epigenetic regulation of microglia plays a vital part in the etiology of ASD (Nardone and Elliott, 2016).

## 6. Conclusion

**Figure 1:**
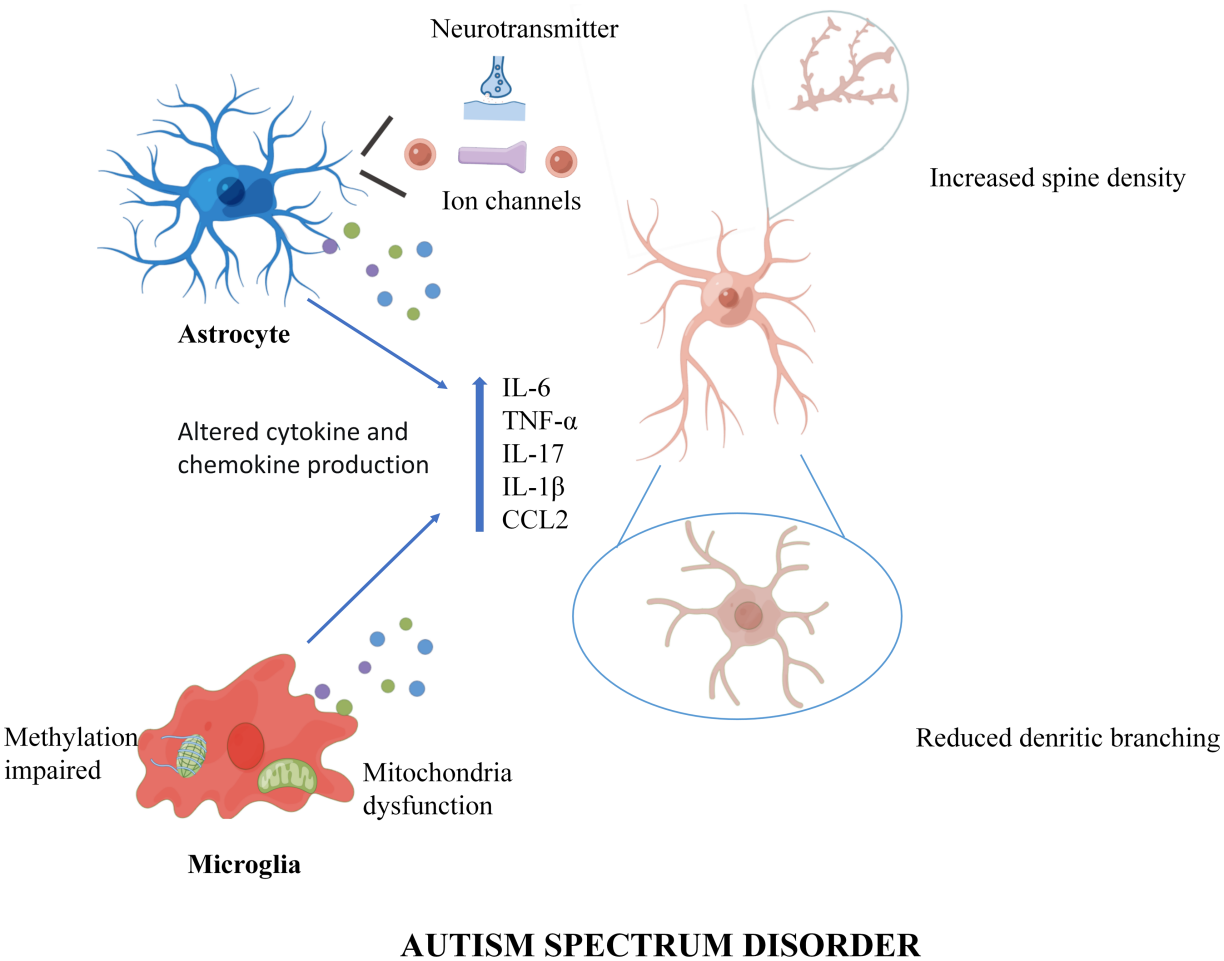
A working model of Microglia and Astrocytes underlie neuroinflammation and synaptic susceptibility in Autism Spectrum Disorder. In ASD, astrocytes enhance synaptic pruning through ion channels and neurotransmitters, while microglia trim synapses through intracellular methylation and mitochondrial alterations. In addition, both microglia and astrocytes can cause neuroinflammation and synaptic changes by releasing inflammatory cytokines and chemokines. Figures were created by Figdraw (www.figdraw.com).

The roles of microglia and astrocytes in ASD were reviewed (Figure 1). Until recently, the role of glial cells was not appreciated in ASD pathogenesis, so neuro-pharmacological strategies to treat symptoms were almost exclusively targeted at neuronal activity and synaptic transmission. First, this review proposes that glial cells could regulate inflammation, synaptic function, and plasticity. In addition, altered neurotransmitters create an abnormal imbalance caused by changes in receptor and transporter expression levels, modification of released glial transmitters, and dysfunction of uptake. Then, the data suggest that glial cell interactions are at least partially involved in the pathogenesis of ASD and that future pharmacological studies should consider improving glial cell functions. In the end, the epigenetics of glial cells should also be considered in the pathogenesis of ASD, suggesting that the study of glial cells may help develop new therapeutic targets for ASD.

## Author contributions

YX and JC designed the study and wrote the manuscript. YL contributed to revising the manuscript. All authors contributed to the article and approved the submitted version.

## Funding

This study was supported by the Natural Science Foundation of Chongqing (No. cstc2021jcyj-msxmX0065).

## Conflict of interest

The authors declare that the research was conducted in the absence of any commercial or financial relationships that could be construed as a potential conflict of interest.

## Publisher’s note

All claims expressed in this article are solely those of the authors and do not necessarily represent those of their affiliated organizations, or those of the publisher, the editors and the reviewers. Any product that may be evaluated in this article, or claim that may be made by its manufacturer, is not guaranteed or endorsed by the publisher.
